# Indicators of Fatigue during a Soccer Match Simulation Using GPS-Derived Workload Values: Which Metrics Are Most Useful?

**DOI:** 10.3390/sports12010009

**Published:** 2023-12-27

**Authors:** Benjamin J. Snyder, Cameron Maung-Maung, Cameron Whitacre

**Affiliations:** 1 Department of Health Sciences, Furman University, Greenville, SC 29613, USA; maunca1@furman.edu; 2 School of Medicine Greenville, University of South Carolina, 607 Grove Rd., Greenville, SC 29605, USA; whitacc@email.sc.edu

**Keywords:** pacing, T-SAFT^90^, high-speed running, accelerations, decelerations, speed intensity, dynamic stress load

## Abstract

Research into women’s competitive soccer matches has shown distance and high-speed running (HSR) reductions over time, but the effects on some GPS-derived metrics have not been investigated. The purpose of this project was to examine the utility of common GPS metrics for indicating fatigue from the T-SAFT^90^ match simulation in collegiate soccer players. Unlike in competitive matches, changes to these metrics occurred as early as 15 min, with HSR, average and max speed, and speed intensity (SI) exhibiting significant declines. HSR and SI were even further decreased in later periods, with HSR lower in minutes 30–40 (T_30–35_ and T_35–40_) than T_15–20_ and lower in T_70–85_ than T_45–60_. SI showed a similar pattern of continued decline, reaching its lowest value in the last three time segments. Accelerations and decelerations were also decreased beginning at T_15–20_ and T_20–25_, respectively, but the fatigue index (FI), dynamic stress load (DSL), and step balance (SB) were unchanged. It can be concluded that in contrast to competitive matches where players can dictate their own intensity, a match simulation may result in a quicker onset of fatigue, but not all GPS-derived values change as expected in fatiguing environments. Coaches and sports scientists may use these findings to properly monitor fatigue in real time.

## 1. Introduction

Soccer is among the most popular sports worldwide and, therefore, one of the most researched. The relatively recent ability of players to wear highly accurate GPS tracking devices during competition has resulted in a large number of studies investigating the physical demands of competitive matches, and the increasing popularity of women’s soccer has prompted a number of studies in that population (see [1] for a comprehensive review). Numerous studies have examined total distance and running intensity in different phases of competitive professional women’s soccer matches. However, far fewer have documented these same changes at the Division I collegiate level [2,3,4,5], none of which have observed changes in segments less than match halves. Of those studies with segmented data, most have found reductions in high-speed running distance and total distance, primarily noted in the last 15 min of matches [6,7,8,9]. Some have also documented changes to accelerations and decelerations at the professional level [10], but no current research has included derived values exclusive to different manufacturers that are often utilized by end users, such as dynamic stress load, speed intensity, and PlayerLoad. Additionally, none of these studies used individualized speed ranges, which have been shown to produce different results than arbitrary speed zones [11].

Given that match fatigue is likely to result in decreased performance, the ability to recognize fatigue during a match or document it after a match would allow coaches to confidently advise players on optimal pacing strategies and make evidence-based changes to personnel. To our knowledge, no studies have tracked complete workload data in collegiate athletes in segments smaller than 45 min, which could be critical to understanding fatigue at this level. This could be due to the unique substitution pattern in collegiate soccer, where players can be substituted in the first half without re-entry and substituted in the second half with one re-entry allowed. In this scenario, players are far less likely to play an entire 90 min, and collecting sufficient workload data for purposes of measuring the effects of fatigue is much more difficult. Therefore, the purpose of the study was to track GPS-derived workload values in 5 min segments during a 90 min match simulation, monitoring changes in running patterns and other metrics that might be useful in detecting match fatigue.

A match simulation was determined to be the best way to collect sufficient data due to the limits imposed by substitution patterns. In addition, since match running is affected by factors such as score [7], player position [8,12], and perhaps volitional pacing [7,13], controlling for distance and prescribing the timing of runs at different speeds would allow for a direct examination of fatigue and workload metrics without the effects of match-related factors.

## 2. Materials and Methods

### 2.1. Participants

Over two seasons, 20 collegiate soccer players with a mean age of 19.9 ± 0.8, competing in Division I soccer in the United States, agreed to participate in the study. Players had just completed a three-month off-season training module consisting of five competitive games.

### 2.2. Study Design

**Figure 1:**
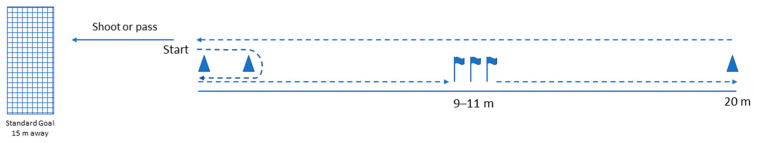
General layout of the T-SAFT^90^ course. Modified from [14].

The study was experimental, with each subject (*n* = 20) serving as their own control during the externally validated technical soccer–specific aerobic field test (T-SAFT^90^), which has been externally validated against match play values. See [14] for more specifics. Workload metrics were tracked in 5 min segments, and verbal, timed commands dictated intensity on a pre-recorded audio track. The simulation was designed to mimic both the aerobic and technical demands of a soccer match. It consists of a 20 m course (Figure 1) that is traversed by participants in response to a series of verbal commands, including “stand”, “walk”, “jog”, “stride”, “sprint”, “dribble”, “jog and shoot”, and “jog and pass”, completed over 90 min with a 15 min half-time break. Subjects travel around a cone 2 m away, returning to the start, then proceeding at the instructed speed to a cone 20 m away, weaving through three poles beginning at 9 m, and returning to the start according to the instructions, which may include a command to shoot or pass into a goal 15 m away. In order to accommodate our measurements of jump parameters every five minutes and to work with our population, we modified the protocol as follows: (1) Rather than being randomized over a 90 min period, the same commands were in the same order for each segment, so that physical demands were identical. (2) The time allowed for each movement was increased by 7%, accounting for the difference in reported top speeds of similar age and skill-level men for whom the simulation was intended [15] while also accounting for the extra rest incurred during the short time in the lab after each 5 min segment, and the number of running tasks reduced to allow the entire protocol to be completed in under 2.25 h. Pilot data confirmed that the intensity was adequate to elicit fatigue but not impossible to maintain. (3) The jump command was removed since jumping was measured on force plates between the 5 min field segments. Over the 90 min, the modified protocol included the same number of shooting, dribbling, and passing tasks as the original T-SAFT^90^ protocol. The expected total distance covered per 5 min segment was 500 m, and the total distance covered was 9000 m.

### 2.3. Methodology

On the day of the test, participants reported to a practice field located adjacent to the human performance lab and completed a comprehensive 15 min warmup similar to the one used before all training sessions. Exercises included jogging, high knees, butt kicks, zig-zag running, shuffling, and sprinting. Participants were then brought into the lab for initial countermovement jump (CMJ) testing consisting of 2–3 maximal jumps. Next, after an explanation and demonstration of the TSAFT^90^, each subject began the simulation. Participants were instructed to adhere to the timing of the tasks as closely as possible, including sprinting maximally with the “sprint” command, and were verbally encouraged throughout the test to ensure compliance with all aspects. After each 5 min segment, participants immediately walked into the adjacent lab. One group (*n* = 8) immediately removed their cleats, put on running flats, and completed two maximal CMJ on a portable force plate (Hawkin Dynamics, Westbrook, ME, USA) that has been validated against a standard in-ground force plate [16]. A second group (*n* = 12) immediately completed the first CMJ in soccer cleats, then put on running flats and completed a second CMJ. Regardless of footwear, the time between entering the lab and returning to the field was between 2 and 2.5 min, and the completion of the jump with running flats occurred at approximately the same time post-run for both groups; thus, the two groups were analyzed together when investigating jump performance with running flats. This process was repeated 18 times with a 15 min “half-time” rest after the ninth repetition.

### 2.4. Workload Parameters

Location, acceleration, direction, and orientation were monitored using a micro-electromechanical device (StatSports Apex, Newry, Northern Ireland, UK), which was mounted in a neoprene pocket affixed between the shoulder blades, reporting GPS data at 18 Hz and accelerometry at 952 Hz. After completion of the match simulation, data were downloaded to a laptop and analyzed using proprietary software (StatSports Apex v. 1.0.01111). Tracked workload parameters are described in Table 1 and include speed-based (individualized high-speed running distance (HSR), speed intensity (SI), and average and maximum speed), accelerometry-based (accelerations (ACC), decelerations (DEC), dynamic stress load (DSL), total loading (TL), and step balance (SB)), physiologic (average heart rate (HR) and heart rate exertion (HRE)), and combined (fatigue index (FI)) measurements. Individualized HSR was based on each participant’s previously determined max speed during a 40-yard maximal sprint [17] and was preferred given the confounding effect of interindividual speed differences on high-speed running data using arbitrary speed zones [11]. In our participants, the average max speed was 27.12 ± 1.75 km/h (7.53 m/s), with a range of 23.2–30.4 km/h (6.44–8.44 m/s). The threshold for HSR was 50% max speed; thus, the mean HSR threshold was 13.55 ± 0.87 km/h.

### 2.5. Countermovement Jump Testing

To prevent slippage, a two-piece portable force plate was covered with a 50 g, 5 mm thick mat. Participants were told to “explode off the platform” with as much force as possible. If the participant lost their balance, landed with part of their foot off the platform, or did not appear to jump maximally, an additional jump was completed to replace the errant one. Force plate data were transmitted via Bluetooth to a handheld tablet (Samsung, Seoul, Republic of Korea) and uploaded automatically to a cloud data site for later analysis. No familiarization jumps were necessary to achieve the best results, as players regularly participated in maximal CMJ sessions as part of their resistance training workouts. Analyzed jump parameters are described in Table 2.

### 2.6. Statistical Analysis

Longitudinal changes were investigated via a linear mixed-model analysis with an autoregressive heterogeneous covariance matrix. Time was the main factor, and fixed effect differences compared to baseline were considered significant at *p* ≤ 0.05. For significant main effects, post hoc analysis was completed via paired *t*-test, and Cohen’s effect sizes (*d*) were calculated, defined as small (*d* < 0.2–0.5), medium (*d* ≥ 0.5, but <0.8), and large (*d* ≥ 0.8, but <1.2), or very large (≥1.2) [18]. All results are presented as mean ± standard deviation (SD).

## 3. Results

### 3.1. Workload Parameters

**Figure 2:**
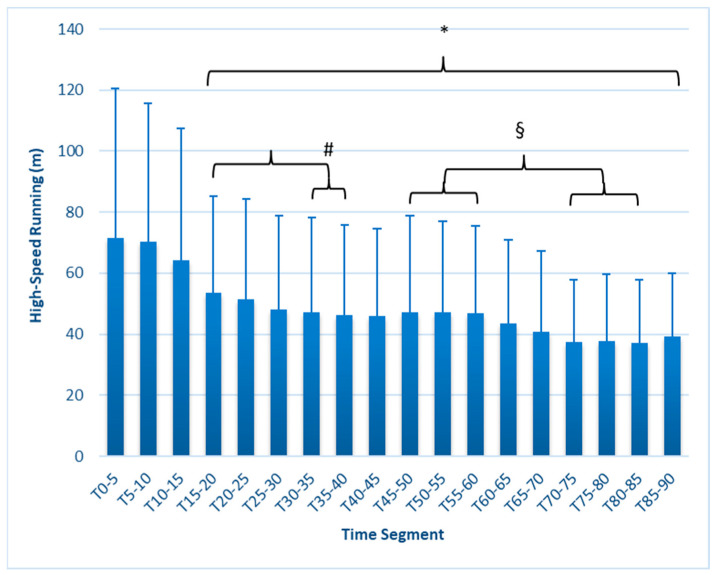
High-speed running (m) in 5 min segments. * Values in brackets sig. different from T_0–5_ (*p* ≤ 0.05). # Values in sub-brackets sig. different from T_15–20_ (*p* ≤ 0.05). § Values in brackets sig. different from each other. Error bars represent SD.

Initial changes to HSR, max speed, average speed, and speed intensity occurred earlier than expected, and some metrics reflected increasing fatigue in later time segments. High-speed running (Figure 2) at T_0–5_ was 62.25 ± 48.2 m but was significantly lower beginning at T_15–20_ (49.09 ± 35 m, *p* = 0.011) and continuing to decrease through most of the test, reaching a low value at T_80–85_ (33.06 m ± 22.1, *p* < 0.001). Values at T_30–35_ (47.3 ± 31.0) and T_35–40_ (46.3 ± 29.5) were significantly less (*p* = 0.025) than T_15–20_, and values between T_70–85_ were significantly lower (*p* = 0.021 or less) than all three-time segments from T_45–60_.

**Figure 3:**
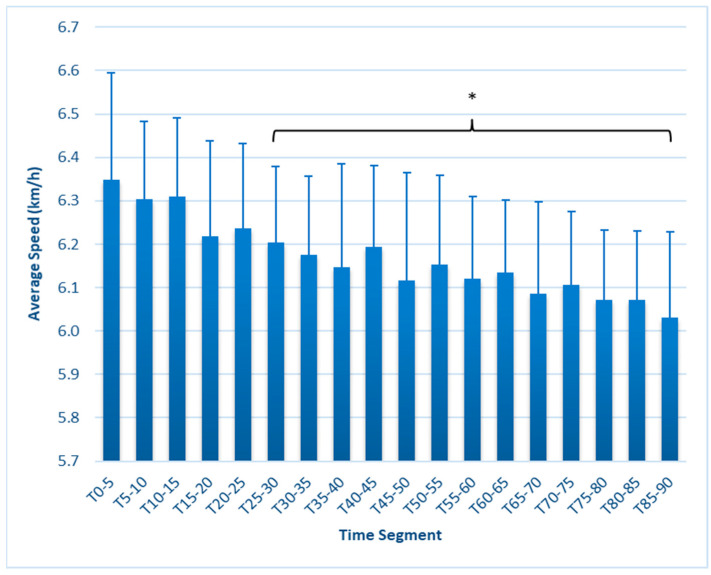
Average speed (km/h) in 5 min segments. * Values in brackets sig. different from T_0–5_ (*p* ≤ 0.05). Error bars represent SD.

**Figure 4:**
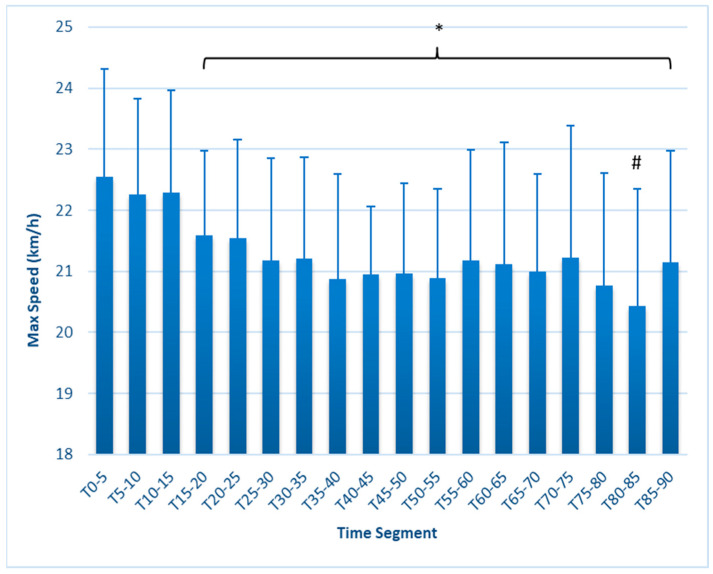
Max speed (km/h) in 5 min segments. * Values in brackets sig. different from T_0–5_ (*p* ≤ 0.05). # Sig. different from segments T_15–25_. Error bars represent SD.

Average speed (Figure 3) was highest at T_0–5_ (6.35 ± 0.25 km/h) and was significantly lower beginning at T_25–30_ (6.205 ± 0.17, *p* = 0.032), remaining lower throughout the test and reaching a nadir in the final time period (6.031 ± 0.20, *p* < 0.001). Max speed (Figure 4) was highest at T_0–5_, at 22.54 ± 1.8 km/h, and was significantly reduced at T_15–20_ (21.58 ± 1.4 km/h, *p* = 0.019), remaining lower throughout the test. The lowest value of 20.43 ± 1.91, found at T_80–85_, represented a further decline from the T_15–20_ (*p* = 0.021) and T_20–25_ (*p* = 0.036) first half values.

**Figure 5:**
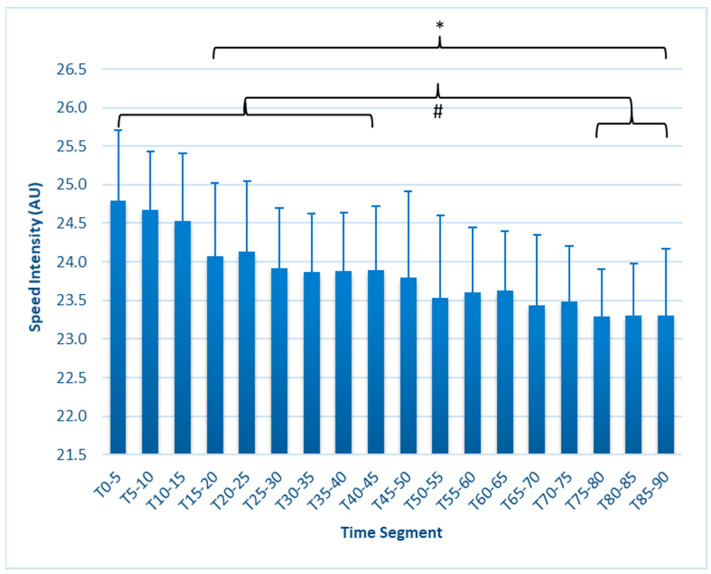
Speed intensity (AU) in 5 min segments. * Values in brackets sig. different from T_0–5_ (*p* ≤ 0.05). # Values in sub-brackets are different from each other. Error bars represent SD.

SI (Figure 5) was reduced from T_0–5_ (24.79 ± 0.92 km/h) beginning at T_15–20_ (24.07 ± 0.96 km/h, *p* = 0.017), remaining lower throughout the test, with segments from T_75–90_ exhibiting further declines from all segments in the first half.

**Figure 6:**
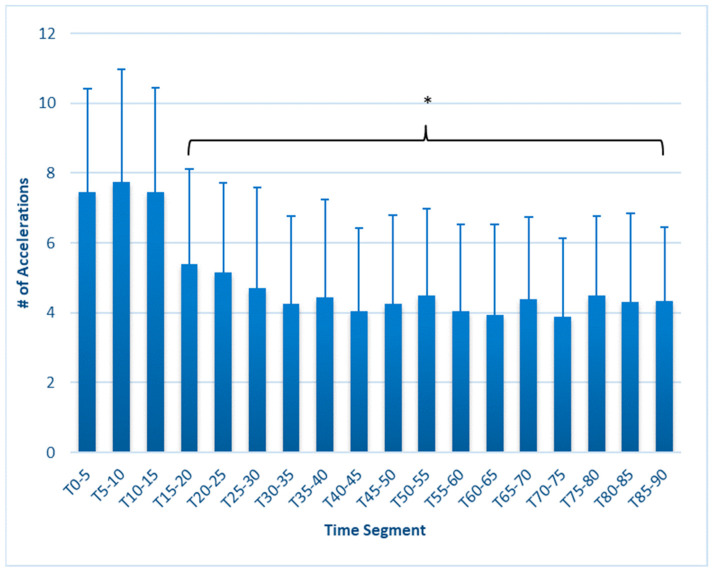
Accelerations in 5 min segments. * Values in brackets sig. different from T_0–5_ (*p* ≤ 0.05). Error bars represent SD.

**Figure 7:**
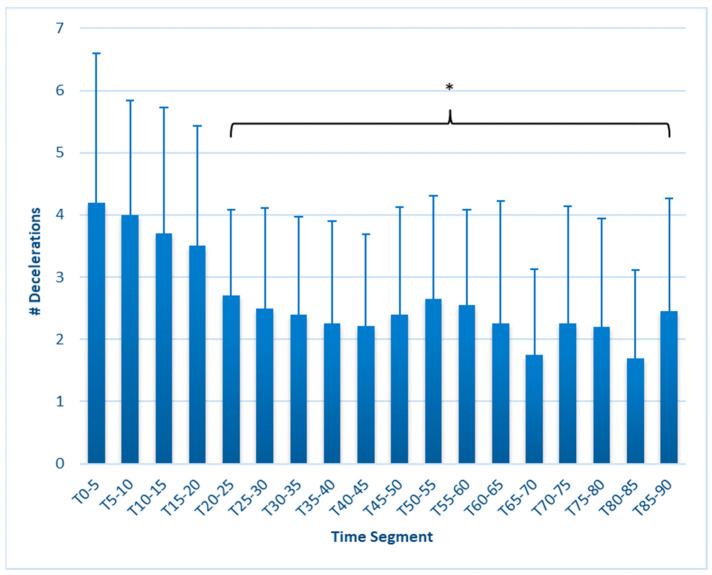
Decelerations in 5 min segments. * Values in brackets sig. different from T_0–5_ (*p* ≤ 0.05). Error bars represent SD.

ACC and DEC both declined, also earlier than expected, but no further changes were seen in later time segments. The ACC (Figure 6) was reduced from T_0–5_ (5.40 ± 2.7) beginning at T_15–20_ (7.45 ± 3.0, *p* < 0.001), remaining lower throughout the test, with a minimum of 3.9 ± 2.3 at segment T_70–75_. DEC (Figure 7) was reduced (*p* = 0.012) at T_20–25_ (2.70 ± 1.4) compared to T_0–5_ (4.2 ± 2.4), remaining lower throughout the test, with a low value of 1.70 ± 1.4 at minutes T_80–85_ (*p* ≤ 0.001).

Total distance traveled averaged 512.98 ± 15.3 m in T_0–5_ and declined slightly over the time course of the test, reaching significance at T_25–30_ (504.54 ± 15.1 m, *p* = 0.023) and remaining lower throughout, with the shortest distance traveled occurring in the final segment (493.76 ± 17.0, *p* < 0.001, *d* = 1.21). These changes were likely due to subjects beginning their decelerations earlier and minimizing extra distance traveled, where, in contrast to the earlier stages, subjects traveled greater than the required total distance of 500 m. The largest change from baseline represented a 3.8% decrease in total distance traveled.

Most changes to workload measurements exhibited moderate to very large effect sizes, summarized in Table 3.

DSL did not show a time effect, remaining steady throughout the test, with a peak mean value of 20.79 ± 2.0 for T_5–10_ and a minimum of 18.3 ± 2.1 for T_65–70_. FI was also unchanged throughout the test, ranging from 0.774 ± 0.38 at T_50–55_ to 0.841 ± 0.38 at minute T_10–15_. TL for T_0–5_ was 7.65 ± 1.2 and was unchanged throughout the test, with the lowest value found at T_80–85_ (7.264 ± 1.26). SB was unchanged throughout the test, remaining well below the 1% difference in load between the left and right foot. Therefore, in the context of a match simulation, these metrics likely do not provide information about player fatigue.

Heart rates during the test were strongly indicative of a high intensity. The average heart rate was lowest at T_0–5_ (167.52 ± 2.1 beats/min) and T_45–50_ (166.61 ± 2.6) due to the effect of low heart rates in the initial 1–2 min from resting values but remained stable throughout the rest of the time periods at around 177 bpm. Heart rate exertion reflected a similar pattern, measuring 18.1 ± 1.3 AU at T_0–5_ and 18.08 ± 1.3 at T_45–50_ but averaging 23.94 ± 1.02 for the rest of the time periods.

### 3.2. Countermovement Jump Parameters

Generally, changes in countermovement jump performance did not exhibit a pattern indicative of persistent fatigue. For jump performance with running flats (*n* = 20), the jump height showed a significant time effect (*p* = 0.011). The lowest jump height was at T_45–50_ (0.286 m ± 0.364), which was significantly lower than T_10–15_, T_15–20_, and T_20–25_ (0.302 ± 0.035, 0.303 ± 0.048, and 0.301 ± 0.040). However, jump height was no different from the pre-test baseline value at any time. The peak relative propulsive power (W/kg) also showed a main effect of time (*p* = 0.008), increasing over the baseline (47.8 ± 6.2) at T_5–10_, T_10–15_, T_15–20_, and T_20–25_ (50.1 ± 6.3, 50.5 ± 7.5, 50.2 ± 6.5, and 49.6 ± 5.9, all *p* < 0.023 or lower). The relative propulsive net impulse showed a significant effect of time (*p* = 0.006), with the lowest value at T_45–50_ (2.38 Ns ± 0.15), which was significantly lower than T_10–15_, T_15–20_, and T_20–25_ (2.44 ± 0.16, 2.45 ± 0.14, and 2.44 ± 0.19). When comparing jump parameters with cleats (*n* = 12), there was a time effect for the propulsive phase (*p* = 0.006), with the lowest value occurring at T_20–25_ (0.221 ± 0.026), which was different from T_0–5_ (0.239 ± 0.028), T_10–15_ (0.236 ± 0.034), T_15–20_ (0.233 ± 0.030), T_40–45_ (0.232 ± 0.023), T_45–50_ (0.238 ± 0.038), T_60–65_ (0.237 ± 0.031), T_70–75_ (0.239 ± 0.031), and T_80–85_ (0.241 ± 0.027), (*p*-values ranging from 0.001 to 0.038) but not different from the baseline. Time to takeoff did not vary from baseline (0.674 ± 0.092) at any time point, but reached a peak at T_40–45_ (0.721 ± 0.137), which was different from T_25–30_ (0.647 ± 0.072) and T_35–40_ (0.659 ± 0.070) (*p* = 0.041 and 0.045). The main effect of time had a *p*-value of 0.002. No other CMJ parameters changed with time. The lack of a fatigue effect may have been due to the slight delay between the end of the previous running segment and the measurement of CMJ, but it may also indicate the general preservation of explosive power.

## 4. Discussion

This study is the first to fully report on changes in external workload metrics during a match simulation in collegiate females. Our findings reveal that in match simulations with externally imposed pacing, significant decreases in speed-related metrics occur as early as 15 min, as measured by average speed, maximal speed, HSR, and SI (Figure 1, Figure 2, Figure 3, Figure 4, Figure 5 and Figure 6). These are unique findings in this population, given that decreases in speed and distance in a competitive match typically occur near the end of the game [6,7,8,9]. At least one study involving competitive matches had similar results to ours. Datson et al. [12] measured running in competitive women’s international matches in 5 min increments and found that although high-speed running was lowest in the last 15 min of the first and second halves, players ran a significantly lower distance at high speed in the 16 to 30 min time period compared to the 0 to 15 min period. These data were obtained from competitive international matches, which may elicit greater high-intensity running than domestic games [6,19], potentially accounting for the early decline. The only similar study employing a match simulation used an equal number of men and women Division I collegiate players, each running at a prescribed intensity corresponding to their fitness level [20]. The physical demands were similar to our study, as their participants completed an average of 9.2 km over the 90 min simulation, compared to our 9050 ± 6.3 m. Measuring running speed, agility, and kinetic parameters such as vertical stiffness and impedance, they found that sprint speed declined at each 7.5 min segment, very similar to our study’s observed change in max speed.

Our participants averaged 876 ± 10.4 m of HSR, using 50% max of individual top speed as measured by a 40 yd sprint. These values are, at first glance, comparable to competitive matches with Division I collegiate players, which ranged from 557 m [5] to approximately 1000 m [21], but since these studies used high-speed running thresholds of 15 km/h, where we used individual thresholds which averaged 13.55 km/h, our participants may have run less than the average high-speed totals of these previous studies.

ACC was decreased from T_0–5_ by 31% at T_20–25_, falling by as much as 47% by T_70–75_, while DEC was significantly lower than T_0–5_ at T_20–25_, a 36% decrease, with a 59% decrease evident by T_80–85_. This might also be an indicator of fatigue, considering the muscular effort required to accelerate (concentric actions) and decelerate (eccentric actions). It is unknown whether this is a consequence of central fatigue or one enforced by alterations to muscle integrity or energy availability. Nonetheless, given the similarity in time profile in ACC and DEC decreases with changes in speed metrics, it seems that ACC/min or DEC/min might be a useful signal of fatigue during a match, although it is possible that match situations would dictate direction changes differently than in a simulation. ACC and DEC are not frequently reported in the literature, and like HSR, thresholds can be varied. Studies of competitive matches using >1 m/s^2^ for ACC ranged from 0.04/min [22] to 0.86/min [23]. The average of 0.987 ACC/min in our population may not be directly comparable since we utilized a >0.5 m/s^2^ threshold. Similarly, our average DEC values of 0.52/min were higher than the 0.23–0.3 decelerations/min reported by Ramos et al. [22], but we used a lower threshold of <−0.5 m/s^2^ in comparison to <−1 m/s^2^ in that study. Two studies of competitive matches by Trewin et al. [24,25] used a higher threshold of 2.26 m/s^2^ and reported significantly higher accelerations per minute, ranging from 1.65 to 1.95 for various positions and situations, but did not report changes over time. However, Mara et al. [10] and Panduro et al. [9] reported a decline in accelerations and decelerations from the first to the second half of competitive matches.

Interestingly, neither DSL nor TL changed during the match simulation, although the literature from the manufacturer describes these metrics as having the potential to measure fatigue or accumulated loading. Hypothetically, as a player tires during a match, their running style might change such that their bodies would absorb greater ground impact forces, resulting in more accumulated “weighted impacts”, but our data did not support such a hypothesis. Fatigue index, which is the ratio of DSL/SI, also did not change despite a 5.9% decrease in SI by T_85–90_, indicating that it might not be sensitive enough to be used to measure fatigue. This suggests that SI on its own would be more useful.

There are some comparable data from match simulation studies in higher-level male players. Huthöfer et al. [26], examining professional male players, measured a change in peak velocity in the 45th and 90th minute compared to the first minute, but no changes were noted in minute 25. This study used a different match simulation, perhaps accounting for the different patterns of change. Another study [27] examined changes to PlayerLoad, a summation of vertical and horizontal forces, during a variable-speed treadmill simulation involving semiprofessional male players, finding an increase at minute 45 compared to the 0–15 and 15–30 min periods. The authors speculated that changes in running efficiency may have contributed to this increase, but the study was conducted on a treadmill, which may have contributed to changes in stride length and, thus, load on the legs. PlayerLoad can best be compared to TL, although they are calculated differently.

The physiologic intensity of our simulation was comparable to or perhaps higher than a typical competitive match. The average heart rate was 178.1 ± 9.5, or approximately 92.3% age-predicted max HR [28], and did not change throughout the test (excluding the first segment of each half when the heart rate had not reached a steady state), indicating a consistent near-maximal effort. By comparison, an examination of elite professional players during competitive matches found that mean HR ranged from 169 bpm in central defenders to 173 bpm in “external midfielders” [9].

In competitive matches, players may engage in a pacing strategy, as described by Edwards and Noakes [13], potentially delaying fatigue, but due to the nature of the match simulation, that was unlikely in our participants. During the test, participants were told to follow the verbal cues as closely as possible, so their speed was largely controlled. Furthermore, each 5 min segment included one maximal sprint, and maximal speed declined significantly by the T_15–20_ (*d* = 0.6), with the lowest value at T_80–85_ representing a 9.3% decrease with a large effect size (*d* = 1.15). Lastly, heart rates in our participants averaged 92% of the estimated max, evidence of near-maximal effort during each segment. It seems likely, then, that fatigue during soccer may be as much a function of intensity over a short period of time as it is of accumulated work. Several studies have shown that an intense 5 min period is followed by a significant drop in running intensity [7,12].

The current study indicates that when the intensity is dictated, rather than being at the player’s discretion, indicators of fatigue occur much earlier, resulting in decreased performance throughout the remaining session. This would seem to indirectly validate the existence and value of pacing in competitive matches.

Jump parameters, including jump height, showed no consistent changes from baseline that would indicate fatigue. This finding is similar to Cone et al. [20], who showed no changes in jump height or ground reaction force of two different types of jumps despite reporting changes in sprint speed over time. Therefore, it seems that the ability to create maximal power is preserved despite other indicators of fatigue. It is possible that the slight time delay (approximately 30 s) between running on the field and jump testing in the lab could have contributed to the lack of change, but since many explosive jumps occur during headers from corners or free kicks, this delay might be game-realistic, mimicking what commonly occurs before set pieces. Future studies of jump performance should attempt to measure jumps within the flow of the running activity, which may limit their relevance in other ways by requiring the entire match simulation to be performed indoors.

In conclusion, a high-intensity match simulation results in changes to high-speed running, max and average speed, speed intensity, accelerations, and decelerations, some of which continue to decline throughout the simulation. In comparison to competitive matches, the externally imposed intensity results in earlier signs of running fatigue, perhaps indirectly confirming a pacing strategy in some competitive matches. While the above metrics might be used as indicators of fatigue during live workload monitoring, some others are not sensitive enough and should be viewed critically for their inclusion in workload analysis. Lastly, changes to countermovement jump parameters were not indicative of fatigue.

## 5. Practical Applications

The finding that match fatigue is present in the first 15 min of a match simulation, where intensity is externally imposed, could be most applicable to a team that chooses to press/counter-press the ball for extended periods during a match, i.e., when the coach is instructing the team to engage in higher intensity activity rather than pacing themselves. It seems that HSR and SI may be useful indicators of fatigue during a live match if they can be tracked on a per-minute or per-segment basis. Likewise, monitoring ACC/min or DEC/min could be useful in this respect. These metrics could also be used in conditioning exercises or high-intensity drills as a measure of fitness or be monitored for improvement over time in similar-intensity sessions. Maximal speed may not occur frequently enough to be of use as a fatigue monitor during a match, while our research has not shown DSL, TL, SB, and FI to be sensitive measures of fatigue.

## Figures and Tables

**Table 1:** Workload metrics—definitions (from manufacturer) and abbreviations.

Value (Unit)	Definition
Accelerations (#)	Number of accelerations of at least 0.5 m/s/s for 0.5 s.
Decelerations (#)	Number of decelerations of at least 0.5 m/s/s for 0.5 s.
Dynamix Stress Load (AU)	Total of weighted impacts above 2 g (scaled).
Fatigue Index (AU)	Dynamic stress load divided by speed intensity.
Heart Rate Exertion (AU)	$C\Sigma W_{i}{dt}_{i}$, where i = 1 to n, the number of time points, C = scaling constant (0.0167), W = heart rate exertion weighting for time point i based on HR/MaxHR, and dt = time interval between successive HR values (0.1 s).
High-Speed Running (meters)	Distance in meters traveled at 50% or greater of previously measured max speed. This variable was customized for the study and varies from software presets.
Speed Intensity (AU)	$\Sigma W_{i}{dt}_{i}$ where i = 1 to n, the number of time points, W = speed intensity weighting for each time point, and dt = time interval between successive speed points (0.1 s).
Step Balance (%)	Ratio of the average peak impact force of the left foot to the sum of the average peak impact of the left and right foot.
Total Loading (AU)	Total of the forces on the subject over the entire session based on accelerometer values in three directions, sampled 100× per second, and scaled by 1000.

AU = Arbitrary units.

**Table 2:** Jump plate metric definitions (from manufacturer).

Value (Unit)	Definition
Jump height	The change in the system center of mass position between the instant of takeoff and peak positive vertical displacement of the system center of mass, calculated using the vertical velocity of the system center of mass at the instant of takeoff and the equations of uniformly accelerated motion.
Countermovement depth	The negative vertical displacement of the system center of mass (m).
Force at minimum displacement	The vertical ground reaction force applied to the system center of mass at the point of peak negative vertical displacement of the system center of mass.
Average relative propulsive force	The average vertical ground reaction force applied to the system center of mass during the propulsion phase as a percentage of system (body) weight.
Peak relative propulsive force	The peak instantaneous vertical ground reaction force applied to the system center of mass during the propulsion phase as a percentage of system (body) weight.
Peak relative propulsive power	The peak instantaneous mechanical power applied to the system center of mass during the propulsion phase relative to system mass (W/kg).
Propulsive phase	The time taken to complete the propulsion phase (from the time when a positive center of mass velocity has been achieved to the moment of takeoff).
Positive impulse	The total vertical impulse applied to the system center of mass during the braking phase and the propulsion phase (Ns).
Time to takeoff	The total time taken from the initiation of movement to the instant of takeoff.
Relative peak landing force	The peak instantaneous ground reaction force applied to the system center of mass during the landing phase (%).
L/R peak landing force	The asymmetry between the left and right vertical ground reaction forces applied to the system center of mass at the instant of peak vertical ground reaction force during the landing phase (%).

**Table 3:** Effect sizes for significant differences (*p* ≤ 0.05) among workload metrics across time segments.

Time Segment/Metric	T_15–20_	T_20–25_	T_25–30_	T_30–35_	T_35–40_	T_40–45_	T_45–50_	T_50–55_	T_55–60_	T_60–65_	T_65–70_	T_70–75_	T_75–80_	T_80–85_	T_85–90_
HSR	0.43	0.48	0.57	0.59	0.62	0.63	0.58	0.59	0.61	0.4	0.77	0.9	0.89	0.91	0.85
Max Speed	NS	0.83	0.94	1.17	1.04	1.27	1.16	1.08	1.24	1.26	1.14	1.35	1.12	1.14	1.2
Average Speed	NS	NS	NS	0.8	0.83	NS	0.93	0.86	1.03	1.02	1.14	1.14	1.33	1.33	1.42
SI	0.77	0.71	1.02	1.1	1.08	1.02	0.97	1.26	1.35	1.37	1.48	1.59	1.92	1.84	1.67
ACC	NS	0.83	0.94	1.17	1.04	1.27	1.16	1.08	1.24	1.26	1.14	1.35	1.12	1.14	1.2
DEC	NS	0.77	0.83	0.89	0.95	1.0	0.86	0.75	0.82	0.89	1.25	0.9	0.96	1.27	0.82

No metrics were significantly different before T_15–20_. NS = non-significant.

## Data Availability

The data presented in this study are only available upon request from the corresponding author. The data are not publicly available due to privacy issues.

## References

[1] Harkness-Armstrong A., Till K., Datson N., Myhill N., Emmonds S. (2022). A systematic review of match-play characteristics in women’s soccer. PLoS ONE.

[2] Bohner J.D., Hoffman J.R., McCormack W.P., Scanlon T.C., Townsend J.R., Stout J.R., Fragala M.S., Fukuda D.H. (2015). Moderate Altitude Affects High Intensity Running Performance in a Collegiate Women’s Soccer Game. J. Hum. Kinet..

[3] Bozzini B.N., McFadden B.A., Walker A.J., Arent S.M. (2020). Varying demands and quality of play between in-conference and out-of-conference games in Division I collegiate women’s soccer. J. Strength Cond. Res..

[4] Vescovi J.D., Favero T.G. (2014). Motion characteristics of women’s college soccer matches: Female Athletes in Motion (FAiM) study. Int. J. Sports Physiol. Perf..

[5] Wells A.J., Hoffman J.R., Beyer K.S., Hoffman M.W., Jajtner A.R., Fukuda D.H., Stout J.R. (2015). Regular- and postseason comparisons of playing time and measures of running performance in NCAA Division I women soccer players. Appl. Physiol. Nutr. Metab..

[6] Andersson H., Raastad T., Nilsson J., Paulsen G., Garthe I., Kadi F. (2008). Neuromuscular Fatigue and Recovery in Elite Female Soccer: Effects of Active Recovery. Med. Sci. Sports Exerc..

[7] Bradley P.S., Noakes T.D. (2013). Match running performance fluctuations in elite soccer: Indicative of fatigue, pacing or situational influences?. J. Sports Sci..

[8] Mara J.K., Thompson K.G., Pumpa K.L., Morgan S. (2017). Quantifying the High-Speed Running and Sprinting Profiles of Elite Female Soccer Players During Competitive Matches Using an Optical Player Tracking System. J. Strength Cond. Res..

[9] Panduro J., Ermidis G., Røddik L.L., Vigh-larsen J.F., Madsen E.E., Larsen M.N., Pettersen S.A., Krustrup P., Randers M.B. (2022). Physical performance and loading for six playing positions in elite female football: Full-game, end-game, and peak periods. Scand. Med. Sci. Sports.

[10] Mara J.K., Thompson K.G., Pumpa K.L., Morgan S. (2017). The acceleration and deceleration profiles of elite female soccer players during competitive matches. J. Sci. Med. Sport.

[11] Abt G., Lovell R. (2009). The use of individualized speed and intensity thresholds for determining the distance run at high-intensity in professional soccer. J. Sports Sci..

[12] Datson N., Drust B., Weston M., Jarman I., Lisboa P., Gregson W. (2017). Match physical performance of elite female soccer players during international competition. J. Strength Cond. Res..

[13] Edwards A.M., Noakes T.D. (2009). Dehydration: Cause of Fatigue or Sign of Pacing in Elite Soccer?. Sports Med..

[14] da Silva C.D., Lovell R. (2020). External Validity of the T-SAFT90: A Soccer Simulation Including Technical and Jumping Activities. Int. J. Sports Physiol..

[15] Mujika I., Santisteban J., Impellizzeri F.M., Castagna C. (2009). Fitness determinants of success in men’s and women’s football. J. Sports Sci..

[16] Badby A.J., Mundy P.D., Comfort P., Lake J.P., McMahon J.J. (2023). The Validity of Hawkin Dynamics Wireless Dual Force Plates for Measuring Countermovement Jump and Drop Jump Variables. Sensors.

[17] Kyprianou E., Di Salvo V., Lolli L., Al Haddad H., Villanueva A.M., Gregson W., Weston M. (2022). To Measure Peak Velocity in Soccer, Let the Players Sprint. J. Strength Cond. Res..

[18] Cohen J. (2009). Statistical Power Analysis for the Behavioral Sciences.

[19] Griffin J., Newans T., Horan S., Keogh J., Andreatta M., Minahan C. (2021). Acceleration and High-Speed Running Profiles of Women’s International and Domestic Football Matches. Front. Sports Act. Living.

[20] Cone J.R., Berry N.T., Goldfarb A.H., Henson R.A., Schmitz R.J., Wideman L., Shultz S.J. (2012). Effects of an Individualized Soccer Match Simulation on Vertical Stiffness and Impedance. J. Strength Cond. Res..

[21] Sausaman R.W., Sams M.L., Mizuguchi S., DeWeese B.H., Stone M.H. (2019). The Physical Demands of NCAA Division I Women’s College Soccer. J. Funct. Morphol. Kinesiol..

[22] Ramos G., Nakamura F.Y., Pereira L.A., Junior W., Mahseredjian F., Wilke C.F., Garcia E., Coimbra C. (2017). Movement Patterns of a U-20 National Women’s Soccer Team during Competitive Matches: Influence of Playing Position and Performance in the First Half. Int. J. Sports Med..

[23] Romero-Moraleda B., Nedergaard N.J., Morencos E., Casamichana D., Ramirez-Campillo R., Vanrenterghem J. (2021). External and internal loads during the competitive season in professional female soccer players according to their playing position: Differences between training and competition. Res. Sports Med..

[24] Trewin J., Meylan C., Varley M.C., Cronin J. (2018). The match-to-match variation of match-running in elite female soccer. J. Sci. Med. Sport.

[25] Trewin J., Meylan C., Varley M.C., Cronin J., Ling D. (2018). The Effect of Match-Factors on the Running Performance of Elite Female Soccer Players. J. Strength Cond. Res..

[26] Huthöfer M., Harbour E., Schwameder H., Kröll J. Sprint mechanical properties of professional soccer players during a match simulation. Proceedings of the International Society of Biomechanics in Sport Conference.

[27] Page R.M., Marrin K., Brogden C.M., Greig M. (2015). Biomechanical and Physiological Response to a Contemporary Soccer Match-Play Simulation. J. Strength Cond. Res..

[28] Tanaka H., Monahan K.D., Seals D.R. (2001). Age-predicted maximal heart rate revisited. J. Am. Coll. Cardiol..

